# Targeted next‐generation sequencing determined a novel *SGCG* variant that is associated with limb‐girdle muscular dystrophy type 2C: A case report

**DOI:** 10.1002/ccr3.7025

**Published:** 2023-03-27

**Authors:** Nam‐Chung Tran, Tuan Anh Nguyen, Thanh Dat Ta, Thinh Huy Tran, Phuoc‐Dung Nguyen, Chi Dung Vu, Van‐Hung Nguyen, The‐Hung Bui, Thanh Van Ta, Van Khanh Tran

**Affiliations:** ^1^ Center for Gene and Protein Research Hanoi Medical University Hanoi Vietnam; ^2^ Hanoi Medical University Hanoi Vietnam; ^3^ University of Medicine & Pharmacy Vietnam National University Hanoi Vietnam; ^4^ Department of Medical Genetics, Metabolism &Endocrinology Vietnam National Children's Hospital Hanoi Vietnam; ^5^ Center for Molecular Medicine, Clinical Genetics Unit, Karolinska Institutet Karolinska University Hospital Stockholm Sweden

**Keywords:** limb‐girdle muscular dystrophy type 2C, sarcoglycanopathies, *SGCG* variant, targeted next‐generation sequencing

## Abstract

Limb‐girdle muscular dystrophy‐type 2C (LGMD2C) is caused by mutations in the *SGCG* gene. Here, we report a case of a 26‐year‐old male who had inactive walking due to proximal muscle weakness. Targeted next‐generation sequencing found a novel variant c.412C > T (Q138*) in the *SGCG* gene.

## 1 INTRODUCTION

Limb‐girdle muscular dystrophies (LGMDs) are a heterogeneous group of rare genetic disorders, characterized by progressive weakness and muscle wasting. Currently, more than 27 different muscular disorders are identified as subtypes of LGMDs. Derived from the inheritance manners, LGMDs were classified into two main types: LGMD type 1 (autosomal dominant: AD) and LGMD type 2 (autosomal recessive: AR).^1^ LGMD2 shows a more common distribution, with a prevalence of 1: 15,000, than LGMD1 (<10% of all LGMD cases).^2^ Moreover, LGMD2 has been investigated to be associated with mutations from a group of sarcoglycanopathies genes such as *SGCG, SGCA, SGCB, SGCD* genes, which cause several subtypes of LGMD2 including LGMD2C to F, respectively.

LGMD2C (OMIM#253700) is initiated by mutations in *SGCG* gene. This gene is located on 13q12, comprising eight exons and encodes for γ‐sarcoglycan. LGMD2C is described by the childhood onset of progressive muscular dystrophy. The average age of onset is 5.3 years, and approximately half of the patients typically lose ambulation at 12 years old. Muscle weakness, calf hypertrophy, high serum CK concentration, respiratory failure and cardiomyopathy are common features.^3^ According to these clinical findings, LGMD2C has been considered a severe childhood autosomal recessive muscular dystrophy (SCARMD) as Duchenne muscular dystrophy (DMD). In Vietnam, the epidemiology of LGMD2C is poorly known and also knowledge about its clinical and genetic features is still limited. Here, we report the clinical, pathologic, and genetic findings of an LGMD2C patient and of some of his family members, who were found to carry known [c.320C > T; p.(Ser107Leu); rs772017929] and novel variant [c.412C > T; p.(Gln138*)] in exon 3 and 4 of *SGCG* gene (NG_008759).

## 2 CASE PRESENTATION

**FIGURE 1:**
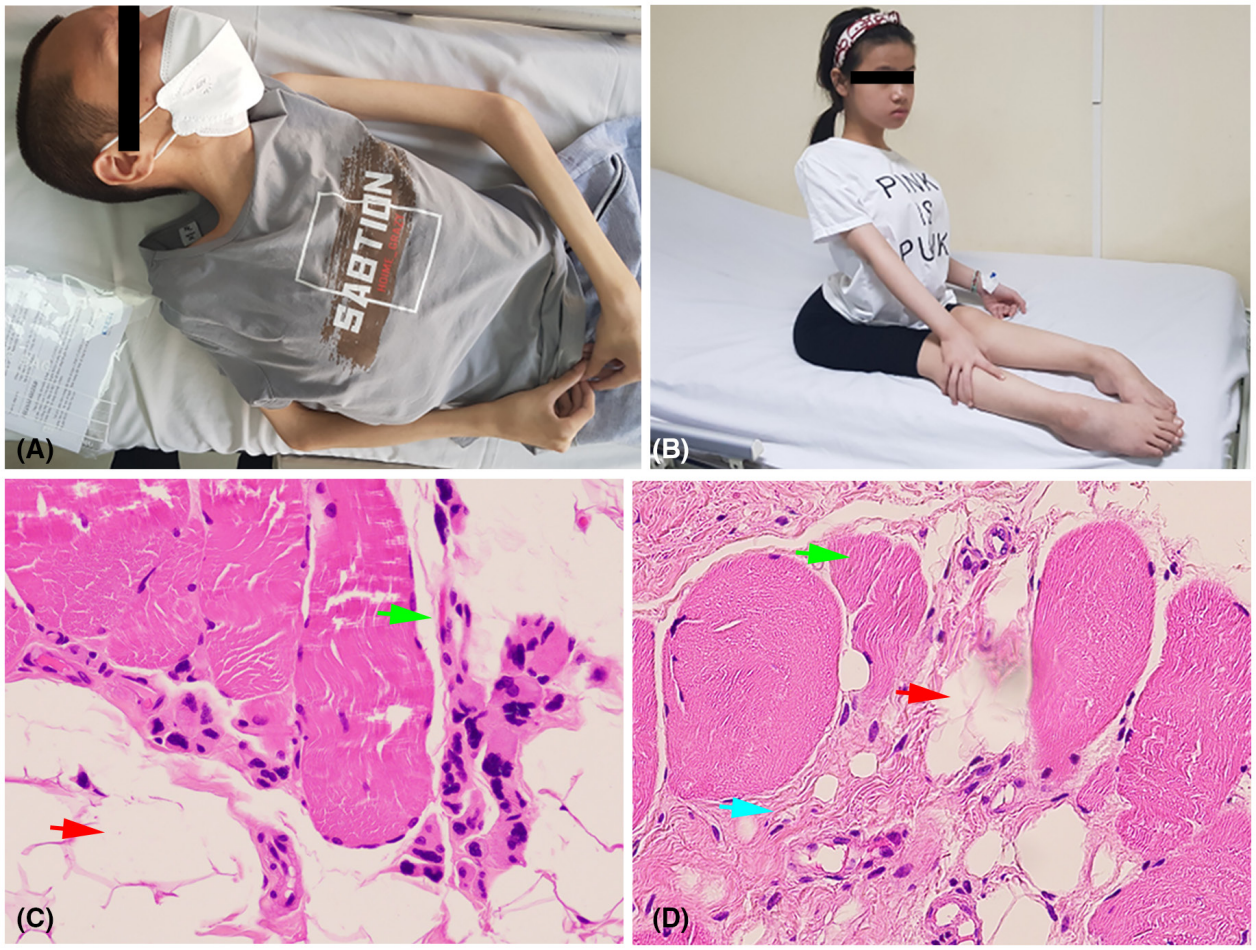
Clinical and histopathological features of LCMD2C. (A) The patient lays on the spot and thinning in shoulders, arms, and thighs. (B) Patient's sister with a slight curvature of the spine. (C) Hematoxylin and eosin staining (HE) at 200× magnification shows degeneration (green arrow) and fatty replacement of muscle fibers (red arrow). (D) Muscle biopsy of sister's patient shows small atrophic fibers (green arrow), fibrosis change (blue arrow), and fatty replacement of muscle (red arrow) (HE, 200×).

A 26‐year‐old male was diagnosed with acute respiratory failure and suspected muscular dystrophy at E Hospital (Hanoi, Vietnam). He was born healthy without any complications. He could walk normally during childhood. When he was 8 years old, the difficulty walking and climbing symptoms appeared and his calves were stiff. At the age of 10, he lost ambulation and lay on the spot until now (Figure 1A). Non‐consanguineous and no history of eventual poliabortivity were recorded in the patient's parents. Physical therapy was used to enhance ambulation and prevent contractures. Muscle biopsy showed the degeneration of muscle fibers and fat infiltration responsible for muscular dystrophy, indicating muscular dystrophy disease (Figure 1C). Among the patient's siblings, his sister (13 years old) carried similar clinical features and was also suspected of muscular dystrophy (Figure 1B); his brother (30 years old) showed no abnormalities. Muscle biopsy of sister's patient showed small atrophic fibers, fibrosis change, and fatty replacement of muscle (Figure 1D). In the electrodiagnostic study, the motor nerve conduction in the peripheral nerves of the extremities was normal. Echocardiogram showed no abnormal findings. His body was thinning in shoulders, arms, and thighs. His muscle showed atrophy in all extremities and calf hypertrophy was not observed. His spine was slightly curvature. Among laboratory tests, serum creatine kinase (CK) increased at 385.8 U/L (reference <171 U/L). His CK‐MB was in the normal range (<24 U/L).

To identify the type of muscular dystrophy, the peripheral blood of the patient and his relatives was collected to extract genomic DNA for genetic analysis, after that the family has provided informed consent. Targeted amplicon sequencing was performed using a gene panel for hereditary muscular dystrophies, on the Illumina HiSeq 2000 platform (Illumina Inc., San Diego, CA). A gene panel was provided in the Supporting Information. Sanger sequencing was performed to confirm the detected abnormalities. To evaluate the pathogenicity of the gene variants disclosed, several bioinformatic tools were applied containing Polyphen‐2: Polymorphism phenotyping 2.0 (http://genetics.bwh.harvard.edu/pph2/), SNPs & GO: GO‐Gene Ontology (https://snps‐and‐go.biocomp.unibo.it/snps‐and‐go/), DUET (DUET (unimelb.edu.au)), Mutation Taster (https://www.mutationtaster.org/), PROVEAN: Protein variation effect analyzer (http://provean.jcvi.org/index.php).

## 3 GENETIC SCREENING

**FIGURE 2:**
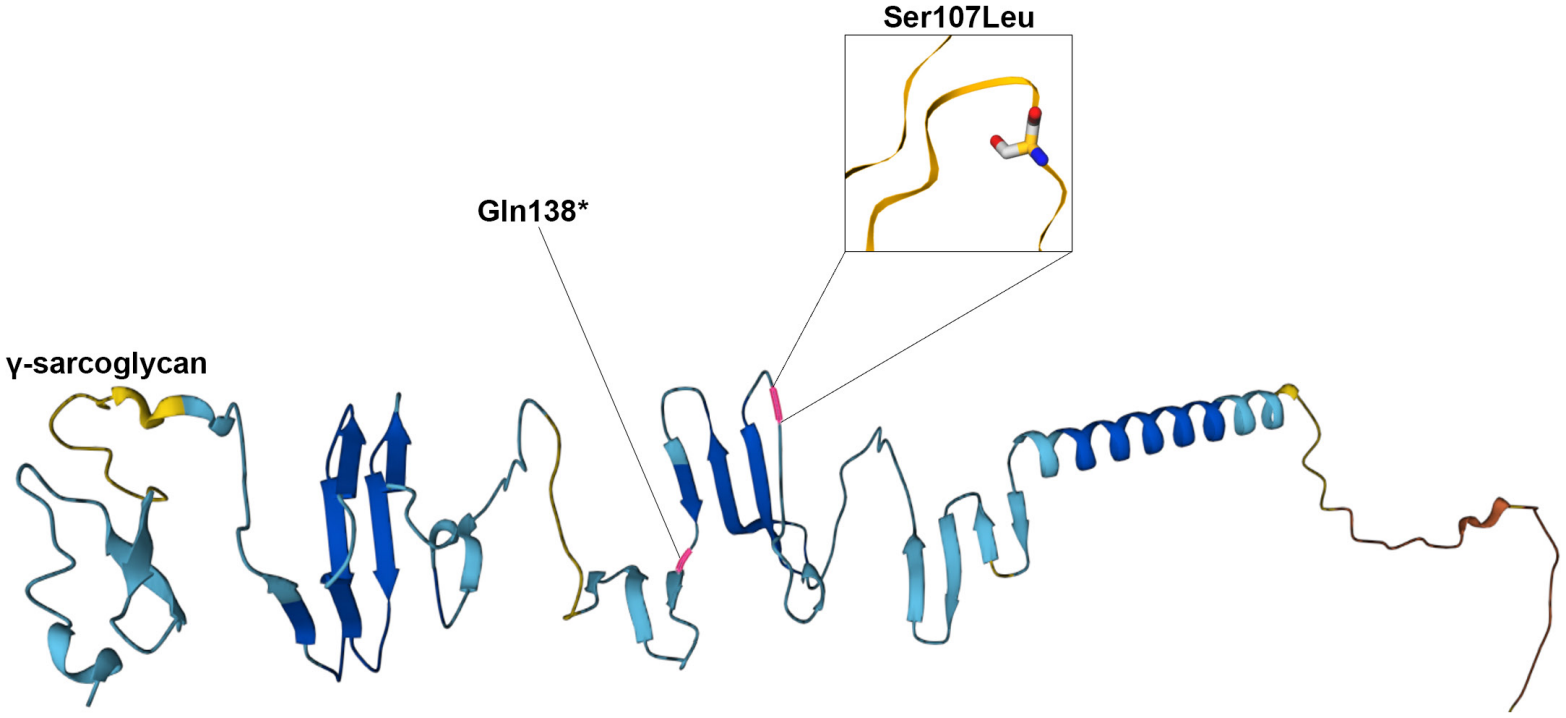
Distribution of two *SGCG* variants on γ‐sarcoglycan protein structure. The model was constructed from UniprotKB (Accession number: Q13326).

**FIGURE 3:**
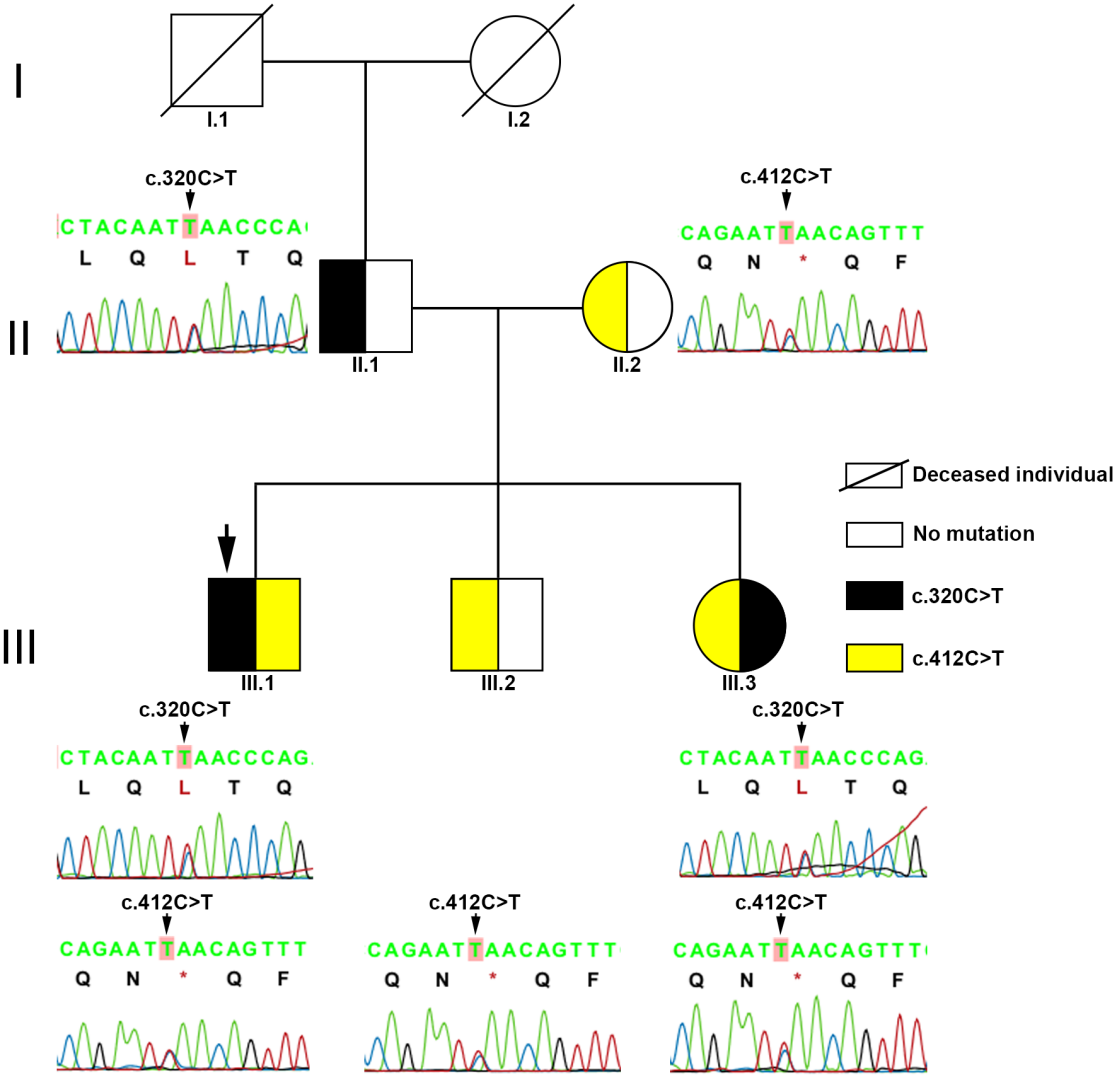
Pedigree of LMGD2C on patient family corresponding to *SGCG* variants. The black arrow shows the patient with LGMD2C (III.1). The red background highlight the altered nucleotide. Black and yellow colors indicate the harboring of *SGCG* variants c.320C > T and c.412C > T, respectively.

Genetic findings of the patient's family indicate the genetic background of the LGMD‐related *SGCG* gene (LGMD2C). As a result, two heterozygous *SGCG* variants were found. The heterozygous C to T base substitution at position 320 in exon 4 of the *SGCG* gene, triggers an amino acid modification from Serine to Leucine at position 107 (NM_000231:c.320C > T, p.Ser107Leu, rs772017929), and a heterozygous C to T base alteration at position 412 in exon 5 of the *SGCG* gene, causing a codon change from Glutamine to a stop codon at position 138 (NM_000231:c.412G > T, p.Gln138*). They were identified as candidate causative mutations (Figure 2). The variant c.412G > T is novel and it has not been recorded before. The patient (III.1) and his sister (III.3) were identified as compound heterozygous of *SGCG* variants c.320C > T (p.Ser107Leu) and c.412C > T (p.Gln138*), and showed an LGMD phenotype. The father (II.1) carried heterozygous *SGCG* variant c.320C > T (p.Ser107Leu); the mother (II.2) and brother (III.2) were heterozygous for c.412G > T (p.Gln138*), healthy and did not show any abnormalities (Figure 3). Unfortunately, we cannot gather samples from the previous generation because all the four grandparents passed away. Moreover, the pathogenicity of two *SGCG* variants was assessed by predictive in silico tools (Table 1). The results indicated that these variants were disease causing, based on nucleotide change and protein structure alteration.

**TABLE 1:** Pathogenic predictive of *SGCG* variants by in silico tools.

Nucleotide alteration	Amino acid alteration	Type of variants	Mutation location	In silico pathogenic analysis				
				Polyphen‐2	SNPs&GO	DUET	Mutation Taster	PROVEAN
c.320C > T	S107L	Missense	Exon 4	Disease‐causing	Disease‐causing	Destabilizing	Disease‐causing	Disease‐causing
c.412C > T	Q138*	Nonsense	Exon 5	N/A	N/A	N/A	Disease‐causing	N/A

*Note*: Disease‐causing: The variant is predicted to cause disease. Destabilizing: The protein structure is predicted to instability caused by variants.

Abbreviations: N/A: Not available.

* The translation stop codon.

## 4 DISCUSSION

In the current study, we conducted the first comprehensive genetic analysis and description of the clinical characteristics of LGMD2C patients in Vietnam. The LGMD2C is evaluated as one of the severe forms of childhood‐onset AR‐LGMD. Although LGMD2C is considered a recessive form of muscular dystrophy as severe as DMD, the inheritance pattern is a clinical hallmark to distinguish them. In addition, among LGMD related to sarcoglycanopathy, the variable expression of sarcoglycan makes them difficult to precisely predict the phenotype.^4^ To accurately diagnose LGMD genotype, next‐generation sequencing (NGS) using a muscular dystrophy gene panel was applied to our patient. NGS strategies have been proven to be an advantageous approach to identify muscular diseases, as electrophysiological investigations or blood chemistry tests are non‐specific.^5^ In addition, NGS is cost‐effective; it allows non‐invasive diagnosis avoiding muscular biopsy.^6^, ^7^, ^8^

We successfully identified two *SGCG* variants: c.320C > T (S107L) and c.412C > T (Gln138*), classifying thus LGMD as type 2C. The *SGCG* gene encodes γ‐sarcoglycan protein which is greatly expressed in skeletal and cardiac muscle. γ‐sarcoglycan belongs to a group of the transmembrane protein linked with the dystrophin‐associated protein (DAP) complex. This complex is mandatory for the stability of muscle membranes and function, and its inhibition results in muscle fiber damage and necrosis.^9^ In our case, *SGCG* variants were found in patient's family including father, mother, brother and sister (Figure 3). However, clinical signs of the disease were absent in father, mother, and brother, as they only carried one heterozygous variant of the *SGCG* gene. The patient and his sister manifested progressive muscular weakness and other LGMD2C clinical features. Their CK levels were 385.8 and 1601.7 U/L, respectively. Also, CK‐MB concentration was 48.4 U/L in the sister (normal range < 24 U/L) and normal in the proband. The significant difference in CK and CK‐MB levels between our patient and his sister may be associated with the advanced stage of LGMD. CK levels in the sister can reflect muscle degeneration at the initial stage of LMGD. Conversely, the lower CK level in the proband may implicate the progression of the dystrophic process at the late stage. Moreover, male patients show more severe and rapid clinical progression than females. The gender differences influencing muscle degeneration can be associated with sex‐related variable hormone levels, such as estrogen and testosterone, and initial muscle mass.^10^, ^11^ Age can negatively induce the biochemical and metabolic alteration in muscle. Older adults tend to degenerate muscle more rapidly than younger ones due to prolonged physical inactivity.^12^ Also, progressive weakness of several respiratory muscles such as diaphragm, intercostal muscles, and accessory respiratory muscles, can lead the respiratory insufficiency.^13^

Of the two identified variants, the missense variant c.320C > T (S107L) (0.00000813 in gnomAD, 0.000008 in ExAC) was the first recorded in the Chinese population; whereas the nonsense variant c.412C > T (Q138*) is a novel variant of *SGCG*.^14^ The common *SGCG* variants c.525delT and c.848G > A, found with high incidence rate (65% in Moroccan and over 40% in Gypsy populations), were not observed in our patient.^15^, ^16^ In silico analysis of two *SGCG* variants here reported have been ascertained to be pathogenic, based on ACMG guidelines. A heterozygous c.320C > T (S107L) and a novel nonsense c.412C > T (Q138*) variant may cause a dysfunctional protein altering its structure. In addition, these variants are localized at the extracellular carboxyl‐terminal of γ‐sarcoglycan (aa 59–291). This domain plays an important role in the retention stability of the sarcoglycan–sarcospan complex (SG‐SSPN).^17^ Without the SG‐SSPN complex, the sarcolemma is very fragile and more vulnerable. In addition, several microdeletions in this domain may cause the loss of γ‐sarcoglycan expression in the muscle. The findings suggest that the extracellular domain plays a critical function in muscle cell survival.

To date, there is no specific treatment for LGMD2C, but different promising approaches, including gene therapy, cell therapy, and pharmacological trials are being investigated.^18^ Furthermore, early diagnosis of LGMD2C can improve the effectiveness of supportive treatments, thus emphasizing the pivotal role of genomic analysis and then also genetic counseling, as well as of prenatal/preimplantation diagnosis. However, the lack of genetic and phenotypic information on LGMD2C in Vietnam is an obstacle to early diagnosis, which actually contributed to the delay in our patient observation, which occurred when he was at the late stage of disease. LGMD2C patients should follow physiotherapy and support mobility treatment with stretch exercises and avoidance of joint contractures. Surgical interventions for orthopedic complications like scoliosis can be required. Cardiological evaluation due to the risk of cardiomyopathy, in addition to respiratory function tests, should also be included in the management of these patients. Follow‐up for cardiomyopathy should be done, although it is uncommon. Baseline cardiac evaluation with echocardiography and respiratory function testing are usual.

## 5 CONCLUSION

Our report described the first case of LGMD2C in Vietnam, providing clinical characteristics, laboratory testing, and genetic findings. We identified a novel variant: c.412C > T (Q138*) of the *SGCG* gene using targeted NGS. An early genomic diagnosis is essential both to limit and slow adverse outcomes and complications and offer adequate counseling, including prenatal/preimplantation diagnosis in case of couples at increased risk. This may allow to enhance the quality of life of patients and of their families, through an individualized approach able to provide the most appropriate therapies of each single case. Further study involving larger cohorts should be performed to clarify the genetic prevalence and the genotype–phenotype correlations of LGMD in the Vietnamese population.

## AUTHOR CONTRIBUTIONS

**Nam‐Chung Tran:** Data curation; formal analysis. **Tuan Anh Nguyen:** Data curation; formal analysis. **Thanh Dat Ta:** Formal analysis; writing – original draft; writing – review and editing. **Thinh Huy Tran:** Methodology. **Phuoc‐Dung Nguyen:** Data curation; formal analysis. **Chi Dung Vu:** Methodology. **Van‐Hung Nguyen:** Data curation. **The‐Hung Bui:** Writing – review and editing. **Thanh Van Ta:** Methodology; supervision; visualization. **Van Khanh Tran:** Methodology; supervision; validation; writing – review and editing.

## FUNDING INFORMATION

None.

## CONFLICT OF INTEREST STATEMENT

The authors declare no conflict of interest.

## CONSENT

Written informed consent was obtained from the patient for publication of this case report details.

## Supporting information


Data S1.

## Data Availability

The data is available from the corresponding author upon request.
