# Supplementary material for: Targeted next‐generation sequencing determined a novel SGCG variant that is associated with limb‐girdle muscular dystrophy type 2C: A case report
Source: Clin Case Rep. 2023 Mar 27;11(3):e7025. doi: 10.1002/ccr3.7025 (PMC10041365; doi:10.1002/ccr3.7025)
Supplement: Supplementary file 1 — Data S1. [file CCR3-11-e7025-s001.docx]

**SUPPLEMENTARY INFORMATION**

**A gene panel for muscular dystrophy diseases**:

| **Hereditary Muscular Disease** | **Gene** |
| --- | --- |
| Duchenne Muscular Dystrophy | *DMD* |
| Becker Muscular Dystrophy | *DMD* |
| Muscular dystrophy-dystroglycanopathy type B1 | *POMT1* |
| Muscular dystrophy-dystroglycanopathy type B2 | *POMT2* |
| Muscular dystrophy-dystroglycanopathy type B4 | *FKTN* |
| Muscular dystrophy-dystroglycanopathy type B5 | *FKRP* |
| Muscular dystrophy-dystroglycanopathy type B6 | *LARGE1* |
| Muscular dystrophy-dystroglycanopathy type B3 | *POMGNT1* |
| Muscular dystrophy-dystroglycanopathy type A7 | *ISPD* |
| Muscular dystrophy-dystroglycanopathy type A5 | *FKRP* |
| Fukuyama congenital muscular dystrophy | *FKTN* |
| Limb-Girdle Muscular Dystrophy type 2C | *SGCG* |
| Limb-Girdle Muscular Dystrophy type 2D | *SGCA* |
| Limb-Girdle Muscular Dystrophy type 2E | *SGCB* |
| Limb-Girdle Muscular Dystrophy type 2F | *SGCD* |
| Limb-Girdle Muscular Dystrophy type 2A | *CAPN3* |
| Limb-Girdle Muscular Dystrophy type 2B | *DYSF* |
| Limb-Girdle Muscular Dystrophy type 2G | *TCAP* |
| Limb-Girdle Muscular Dystrophy type 2H | *TRIM32* |
| Limb-Girdle Muscular Dystrophy type 2I | *FKRP* |
| Limb-Girdle Muscular Dystrophy type 2K | *POMT1* |
| Limb-Girdle Muscular Dystrophy type 2J | *TTN* |
| Limb-Girdle Muscular Dystrophy type 2L | *ANO5* |
| Limb-Girdle Muscular Dystrophy type 2M | *FKTN* |
| Limb-Girdle Muscular Dystrophy type 2N | *POMT2* |
| Limb-Girdle Muscular Dystrophy type 2O | *POMGNT1* |
| Limb-Girdle Muscular Dystrophy type 2Q | *PLEC* |
| Limb-Girdle Muscular Dystrophy type 2R | *DES* |
| Limb-Girdle Muscular Dystrophy type 2S | *TRAPPC11* |
| Limb-Girdle Muscular Dystrophy type 1A | *MYOT* |
| Limb-Girdle Muscular Dystrophy type 1B | *LMNA* |
| Limb-Girdle Muscular Dystrophy type 1C | *CAV3* |
| Limb-Girdle Muscular Dystrophy type 1D | *DES* |
| Limb-Girdle Muscular Dystrophy type 1E | *DNAJB6* |
| X-linked Emery-Dreifuss Muscular Dystrophy 1 | *EMD* |
| X-linked Emery-Dreifuss Muscular Dystrophy 6 | *FHL1* |
| Emery-Dreifuss muscular dystrophy 2 | *LMNA* |
| Emery-Dreifuss muscular dystrophy 3 | *LMNA* |
| Emery-Dreifuss muscular dystrophy 4 | *SYNE1* |
| Emery-Dreifuss muscular dystrophy 5 | *SYNE2* |
| Emery-Dreifuss muscular dystrophy 7 | *TMEM43* |
| Congenital myasthenic syndrome with tubular aggregates 2 | *DPAGT1* |
| AGRN-Related Congenital Myasthenic Syndrome | *AGRN* |
| CHAT-Related Congenital Myasthenic Syndrome | *CHAT* |
| CHRNA1-Related Congenital Myasthenic Syndrome | *CHRNA1* |
| CHRNB1-Related Congenital Myasthenic Syndrome | *CHRNB1* |
| CHRND-Related Congenital Myasthenic Syndrome | *CHRND* |
| CHRNE-Related Congenital Myasthenic Syndrome | *CHRNE* |
| COLQ-Related Congenital Myasthenic Syndrome | *COLQ* |
| DOK7-Related Congenital Myasthenic Syndrome | *DOK7* |
| GFPT1-Related Congenital Myasthenic Syndrome | *GFPT1* |
| MUSK-Related Congenital Myasthenic Syndrome | *MUSK* |
| RAPSN-Related Congenital Myasthenic Syndrome | *RAPSN* |
| SCN4A-Related Congenital Myasthenic Syndrome | *SCN4A* |
| Hyperkalemic Periodic Paralysis | *SCN4A* |
| Hypokalemic Periodic Paralysis | *CACNA1S、SCN4A* |
| Myofibrillar Myopathy 1 | *DES* |
| CRYAB-related Myofibrillar Myopathy | *CRYAB* |
| Myofibrillar Myopathy 3 | *MYOT* |
| Myofibrillar Myopathy 4 | *LDB3* |
| Myofibrillar Myopathy 5 | *FLNC* |
| Myofibrillar Myopathy 6 | *BAG3* |
| FHL1-Related Myofibrillar Myopathy | *FHL1* |
| DNAJB6-Related Myofibrillar Myopathy | *DNAJB6* |
| Autosomal Recessive Progressive external ophthalmoplegia | *POLG* |
| Adenosine Monophosphate Deaminase Deficiency | *AMPD1* |
| Danon disease | *LAMP2* |
| Pseudocholinesterase deficiency | *BCHE* |
| X-Linked Dystonia-Parkinsonism | TAF1 |
